# Inhibition of complement C5a prevents breakdown of the blood-brain barrier and pituitary dysfunction in experimental sepsis

**DOI:** 10.1186/cc7710

**Published:** 2009-02-06

**Authors:** Michael A Flierl, Philip F Stahel, Daniel Rittirsch, Markus Huber-Lang, Andreas D Niederbichler, L Marco Hoesel, Basel M Touban, Steven J Morgan, Wade R Smith, Peter A Ward, Kyros Ipaktchi

**Affiliations:** 1 Department of Orthopaedic Surgery, Denver Health Medical Center, University of Colorado School of Medicine, 777 Bannock Street, Denver, CO 80204, USA; 2 Department of Trauma Surgery, University Hospital Zurich, Rämistrasse 100, 8091 Zurich, Switzerland; 3 Department of Trauma, Hand-, Plastic-, and Reconstructive Surgery, University Hospital Ulm, Steinhövelstrasse 9, 89075 Ulm, Germany; 4 Department of Plastic, Hand, and Reconstructive Surgery, University Medical Center Hannover (MHH), Carl-Neuberg-Strasse 1, 30625 Hannover, Germany; 5 Department of Pathology, University of Michigan Medical School, 1301 Catherine Road, Ann Arbor, MI 48109, USA

## Abstract

**Introduction:**

Septic encephalopathy secondary to a breakdown of the blood-brain barrier (BBB) is a known complication of sepsis. However, its pathophysiology remains unclear. The present study investigated the effect of complement C5a blockade in preventing BBB damage and pituitary dysfunction during experimental sepsis.

**Methods:**

Using the standardised caecal ligation and puncture (CLP) model, Sprague-Dawley rats were treated with either neutralising anti-C5a antibody or pre-immune immunoglobulin (Ig) G as a placebo. Sham-operated animals served as internal controls.

**Results:**

Placebo-treated septic rats showed severe BBB dysfunction within 24 hours, accompanied by a significant upregulation of pituitary C5a receptor and pro-inflammatory cytokine expression, although gene levels of growth hormone were significantly attenuated. The pathophysiological changes in placebo-treated septic rats were restored by administration of neutralising anti-C5a antibody to the normal levels of BBB and pituitary function seen in the sham-operated group.

**Conclusions:**

Collectively, the neutralisation of C5a greatly ameliorated pathophysiological changes associated with septic encephalopathy, implying a further rationale for the concept of pharmacological C5a inhibition in sepsis.

## Introduction

Sepsis remains a leading cause of morbidity and mortality in the intensive care unit (ICU), and one of the top 10 causes of death worldwide [1,2]. The underlying pathophysiological cascade of sepsis is highly complex and far from fully understood [3-5]. From an immunological standpoint, the activation of the complement cascade, a potent arm of the innate immune response, has been associated with fatal outcomes in septic patients [6-9]. Particularly, the complement anaphylatoxin C5a, a small inflammatory peptide derived from complement activation, has been characterised as a 'key' mediator of sepsis and septic organ dysfunction [10-14], and was recently labelled as 'too much of a good thing' or to reveal 'a dark side in sepsis' [15,16].

Although intentionally beneficial, disproportionate activation of complement during sepsis has been found to contribute to thymocyte and adrenomedullary apoptosis [17,18], paralysis of innate immunity [19,20], deterioration of the coagulation/fibrinolytic system [21] and multiple organ failure [22]. Accordingly, blockade of C5a or its receptors has been shown to prevent multiple organ failure and to greatly attenuate mortality after caecal ligation and puncture (CLP)-induced sepsis [10,11,14,19,22].

Encephalopathy syndrome is a well described complication of sepsis in the ICU. This phenomenon is thought to represent a consequence of inflammation-mediated dysfunction of the blood-brain barrier (BBB), thus allowing neurotoxic mediators to extravasate from the peripheral circulation into the subarachnoid space or into the brain parenchyma. Noteworthy, the focus of research studies have only addressed in more depth the neuro-inflammatory and metabolic intracerebral changes in sepsis [23-29]. The complement anaphylatoxin C5a has been characterised as a mediator of BBB dysfunction in a variety of central nervous system (CNS) disorders, including traumatic brain injury and bacterial meningitis [30-32]. In addition, the detection of the C5a receptor (C5aR) on neurons and the observed upregulation of neuronal C5aR expression under inflammatory conditions [31,33-35] renders the brain more vulnerable to C5a-mediated neuropathophysiological sequelae secondary to a disruption of the BBB [30,31,36,37]. The complement cascade has only recently been implicated in the pathophysiology of septic encephalopathy [38].

Based on the established functions of C5a in the pathophysiology of sepsis and on experimental data which imply C5a is a potent mediator of BBB damage and neuroinflammation, we designed the present study to investigate the effect of antibody-mediated C5a-blockade on preventing the development of encephalopathy in experimental sepsis. We hypothesised that blockade of C5a would reverse the dysfunction of the BBB and restore the immunological and endocrinological homeostasis in the septic brain.

## Materials and methods

### Experimental CLP model

All procedures were performed in accordance with the National Institutes of Health guidelines and University Committee on Use and Care of Animals, University of Michigan (UCUCA approval #8575, 08/11/2008). Specific pathogen-free, adult male Sprague-Dawley rats (Harlan Inc., Indianapolis, IN, USA) weighing 300 to 350 g were used in all experiments. Sepsis was induced by the CLP procedure as previously described [39]. In brief, rats were anaesthetised with isoflurane (3%, oxygen flow 3 L/minute). After abdominal midline incision, the caecum was exposed, ligated and punctured through with a 18-gauge needle, and a small portion of faeces was expressed to ensure potency of the punctures. After repositioning of the bowel, the abdomen was closed in layers using 4-0 surgical sutures (Ethicon Inc., Somerville, NJ, USA) and metallic clips. Sham animals underwent the same procedure except for ligation and puncture of the caecum. Before and after the surgery, animals had unrestricted access to food and water. Where indicated, animals intravenously received 500 μg anti-C5a antibody or 500 μg preimmune immunoglobulin (Ig) G in 500 μl sterile Dulbecco's PBS immediately after CLP or sham procedure, as previously described [13].

### Anti-C5a antibody

The neutralising anti-rat C5a antibody used in this study was previously characterised [10,22]. In short, rat C5a peptide corresponding to amino acid residues 17 to 36 was coupled to keyhole limpet haemocyanin and used as an antigen to immunise rabbits. After several immunisations, the antibody was affinity purified from serum using the synthetic peptide coupled to beads. Cross-reactivity with recombinant rat C5a was confirmed by Western blotting.

### Albumin immunohistochemistry

Rat brains were surgically removed, embedded in optimal cutting temperature compound (Miles, Elkhart, IN, USA) and stored at -80°C. Frozen sections (10 μm) were prepared from the embedded tissue and incubated with rabbit anti-rat albumin antibody (Bethyl Laboratories, Montgomery, TX, USA) at a dilution of 1/100 overnight. After washing, sections were incubated with a 1/200 dilution of peroxidase-conjugated goat anti-rabbit IgG for two hours (Jackson ImmunoResearch Laboratories, West Grove, PA, USA). After thorough washing, sections were stained using the ImmPACT DAB staining kit (Vector Laboratories, Burlingame, CA, USA). Tissue sections were then mounted with Crystal Mount mounting medium (Sigma, St. Louis, MO, USA) and addition of coverslips. Staining was assessed using light microscopy (BX41, Olympus, Center Valley, PA, USA) and digital imaging (Adobe Photoshop, Adobe, San Jose, CA, USA). For each experimental condition, three animals were used. The immunostainings displayed are representative for three independent experiments.

### Intracerebral Evans Blue assessment

The extent of BBB dysfunction was assessed 24 hours after induction of CLP by assessment of Evans Blue (EB) extravasation in four animals per experimental condition. Briefly, 20 μl of a 2% solution of EB in saline was injected into the tail vein one hour before harvesting of brains (i.e. at t = 23 hours after CLP), and allowed to circulate for 60 minutes. Subsequently, the chest was surgically opened under anaesthesia and the intravascular dye was removed by saline perfusion (40 to 50 ml) through the left heart ventricle. The brain/pituitary was then removed and weighed before homogenisation and placed in 4 mL 99.5% formamide per gram of tissue in polyethylene tubes (BD Bioscience, Rockville, MD, USA). Tubes were placed for 48 hours on a shaker (Barnstead International, Dubuque, IA, USA) at room temperature for EB extraction. Supernatants were collected and the absorbance read in a plate reader (Biotek Instruments, Winooski, VT, USA) at 620 nm and compared with an EB standard curve and formamide blanks. The result are expressed as microgram EB per milligram tissue.

### Isolation of total RNA and quantitative real-time PCR

Total RNA was isolated from five to seven pituitary glands per experimental condition using the TRIzol method (Life Technologies Inc., Gaithersburg, MD, USA) according to the manufacturer's instructions. Digestion of any contaminating DNA was achieved by treatment of samples with RNase-Free DNase (Promega Corp., Madison, WI, USA). Reverse transcription was performed with 5 μg RNA using the Superscript II RNase H^- ^Reverse Transcriptase (GIBCO BRL; Life Technologies Inc., Gaithersburg, MD, USA) according to the manufacturer's protocol. Real-time PCR was then performed as previously described [13]. Reactions were prepared in duplicates using the iQ SYBR green Supermix reagent (Bio Rad Laboratories, Hercules, CA, USA). An amplification plot was generated using two-fold dilutions of the cDNA generated from a known amount (1 μg) of mRNA. The cycle threshold (C_T_) was set above the baseline fluorescence. Plotting the log of the dilutions versus the C_T _values then generated a standard curve. Quantitation of samples was determined using the standard curves. Genes analysed were C5aR, the adrenocorticotropic hormone (ACTH)-precursor proopiomelanocortin (POMC) and growth hormone (GH).

The following primer pairs were used:

*C5aR:*5' primer, 5'-TATAGTCCTGCCCTCGCTCAT-3'; 3' primer, 5'-TCACCACTTTGAGCGTCTTGG-3'.

*POMC:*5' primer, 5'-AGCCTCTGTCCAGTCCTGAG-3'; 3' primer, 5'-CTTAGTCACTGCTCCTTAAC-3'.

*GH*: 5' primer, 5'-CTCGGACCGCGTCTATGAGA-3'; and 3' primer, 5'-TGAGGATCTGCCCAATACGG-3'.

*TNF*: 5' primer, 5'-GTGATCGGTCCCAACAAGGA-3'; and 3' primer, 5'-AGGGTCTGGGCCATGGAA-3'.

*IL-6*: 5' primer, 5'-ATATGTTCTCAGGGAGATCTTGGAA-3'; and 3' primer, 5'-GTGCATCATCGCTGTTCATACA-3'.

*GAPDH: *5' primer, 5'-GCCTCGTCTCATAGACAAGATG-3'; and 3' primer, 5'-CAGTAGACTCCACGACATAC-3'.

### Western blot analysis of C5aR

Following decapitation, rat brains were immediately removed surgically, the pituitary identified and excised, homogenised in ice-cold radio immunoprecipitation assay (RIPA) buffer (Upstate, Temecula, CA, USA) and subjected to BCA Protein Assay analysis (Thermo Scientific, Rockford, IL, USA) for equal protein loading. Total protein from pituitary lysates (50 μg) was separated by electrophoresis in a denaturing 12% polyacrylamide gel and then transferred to a polyvinylidene fluoride membrane. Equal loading was confirmed by detection of glyceraldehyde 3-phosphate dehydrogenase (GAPDH) (Santa Cruz, Santa Cruz, CA, USA) as a 'housekeeping' protein. Non-specific binding sites were blocked with Tris-buffered saline Tween-20 (TBST) plus 5% nonfat dry milk for one hour at room temperature. Following washing in TBST, the membrane was incubated with rabbit anti-rat C5aR antibody (kindly provided by P.A. Ward, University of Michigan [14,40,41]) at a 1/500 dilution overnight. After three washes in TBST, the membrane was incubated in a 1/10,000 dilution of horseradish peroxidase-conjugated donkey anti-rabbit IgG as the secondary antibody (Amersham, Piscataway, NJ, USA). The membrane was developed by enhanced chemiluminescence technique according to the manufacturer's protocol (Amersham, Piscataway, NJ, USA). Pituitary tissue was harvested from five animals and assessed by Western blotting. Due to the lane restrictions (n = 10) of a typical Western mini gel, two (sham groups) or three (CLP groups) samples were compared, in order to be able to evaluate samples directly 'side-by-side'. The blot depicted is representative of three independent experiments.

### ELISA of pituitary hormone levels

Rat whole blood was collected into syringes containing anticoagulant citrate dextrose (Baxter, Deerfield, IL, USA) in a 9:1 ratio by puncture of the inferior vena cava 24 hours after CLP or sham surgery. Samples were centrifuged (2500 rpm for 10 minutes at 4°C), plasma was obtained and immediately stored at -80°C. Levels of GH and corticosterone were determined using commercially available ELISA kits (both Diagnostic Systems Laboratories, Webster, TX, USA) according to the manufacturer's instructions. For plasma measurements of GH and corticosterone, five to seven samples were assessed for each experimental group.

### Statistical analysis

All values are expressed as mean ± standard deviation. Data were analysed with a one-way analysis of variance, and individual group means were then compared with the Tukey multiple comparison test. Differences were considered statistically significant at p < 0.05.

## Results

### Anti-C5a prevents BBB breakdown during experimental sepsis

**Figure 1:**
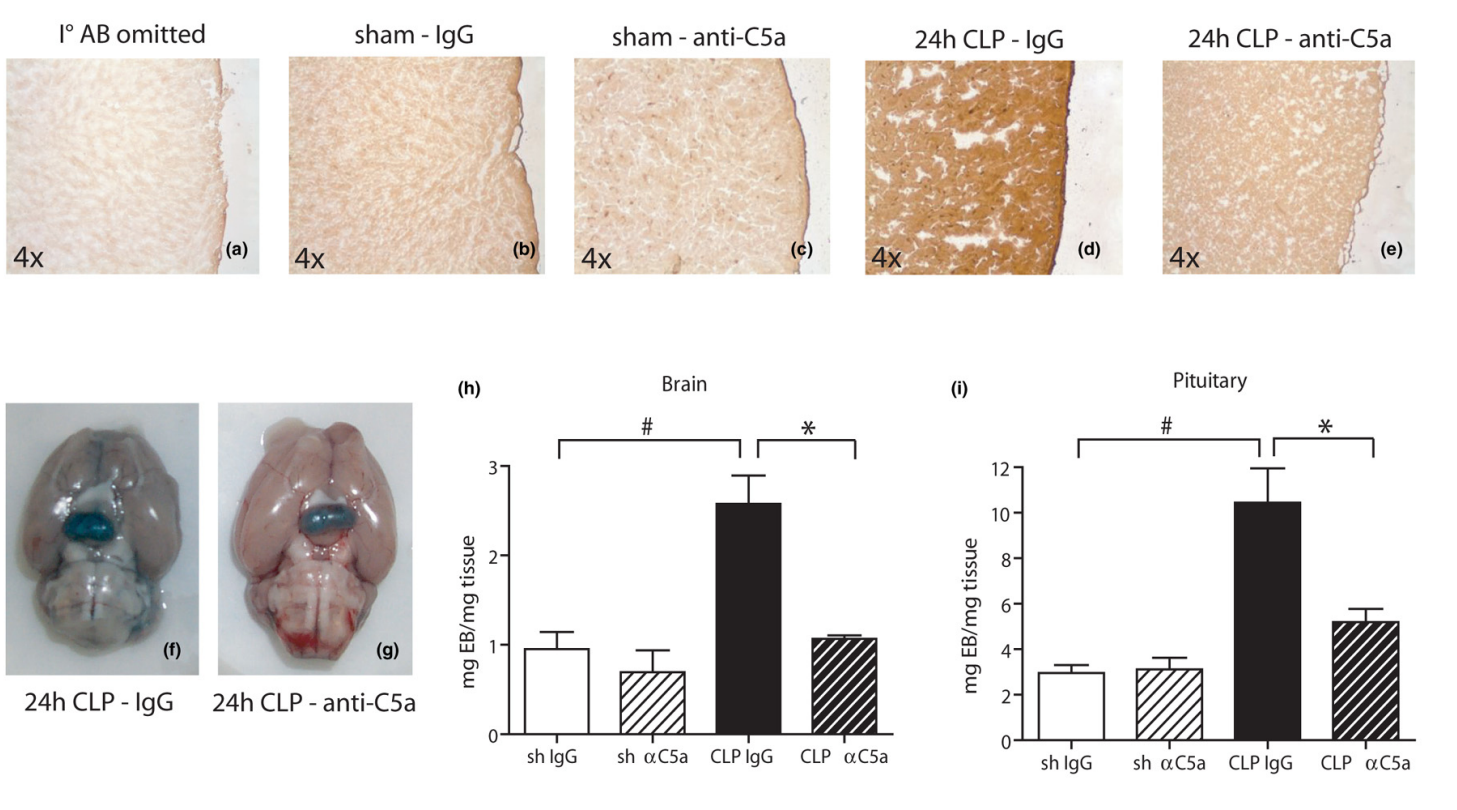
Anti-C5a ameliorates impairment of the blood-brain barrier after caecal ligation and puncture (CLP). **(a-e) **Brains were surgically removed, snap-frozen and tissue sections (10 μm) were obtained. Cerebral extravasation of rat albumin was assessed by immunohistochemistry 24 hours after CLP or sham procedure, three samples per experimental condition. Stains displayed are representative of three independent experiments. **(f, g) **Comparison of Evans Blue extravasation into the cerebellum and pituitary in IgG-treated or anti-C5a treated rats 24 hours post CLP. Displayed depictions are representative of four animals. **(h, i) **Quantification of Evans Blue extravasation into the brain or pituitary by determination of mg Evans Blue/mg tissue ratio in different groups at indicated time-points, four for each experimental condition. # p < 0.05 between sham and 24 hours CLP animals; * p < 0.05 between IgG-treated and anti-C5a-treated rats.

As depicted in Figure 1, as a negative control, primary antibody was omitted in a section obtained from a preimmune IgG-treated animal (panel a) and little straining of brain tissue for albumin was noted. Sham animals treated with either preimmune IgG (panel b) or anti-C5a (panel c) displayed comparable levels of baseline immunostaining for albumin. However, there was a significant increase in diffuse cerebral albumin accumulation 24 hours after CLP in animals treated with preimmune IgG (panel d). In contrast, when rats received anti-C5a immediately after the CLP procedure, cerebral albumin build-up was dramatically reduced to sham levels (panels b and e).

Similar results were found when breakdown of the BBB was analysed by EB extravasation 24 hours after CLP. Animals treated with preimmune IgG displayed robust EB extravasation in the cerebral and pituitary areas (panel f), while anti-C5a-treated littermates exhibited far less buildup of EB (panel g). Panels h and i show quantified EB extravasation 24 hours after CLP as mg EB/mg tissue ratio and confirm the observations made in panels f and g on a quantitative level.

### C5aR is upregulated in the pituitary gland of septic rats

**Figure 2:**
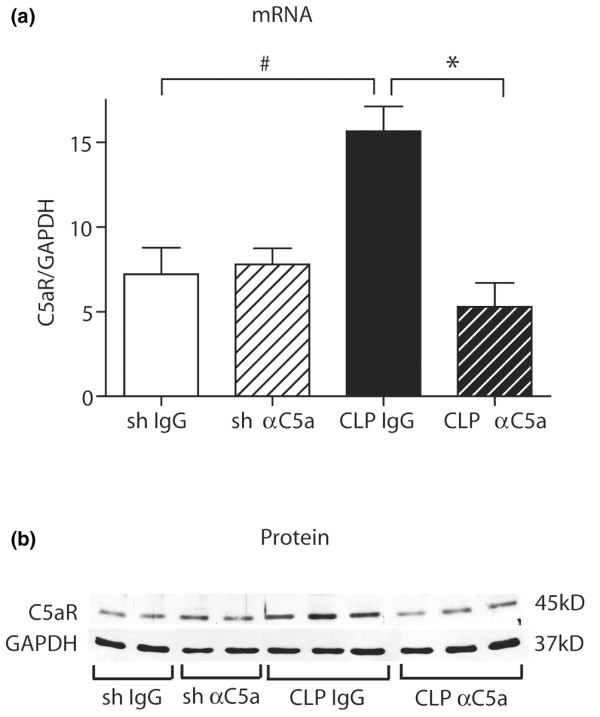
Pituitary expression of C5a receptor (R) during experimental sepsis in IgG or anti-C5a IgG treated sham animals and septic littermates 24 hours after caecal ligation and puncture (CLP) procedure. **(a) **Following total RNA isolation from pituitary tissue, pituitary C5aR mRNA expression was assessed by real-time PCR. For each bar, sample size was five to seven. **(b) **Five pituitary tissue samples were removed at indicated time-points, homogenised and C5aR protein expression was analysed by Western blotting. For sham groups, two samples were taken; for CLP groups, three samples were taken. Blot is representative for three independent experiments. GAPDH = glyceraldehyde 3-phosphate dehydrogenase. # p < 0.05 between sham and 24 hours CLP animals; * p < 0.05 between IgG-treated and anti-C5a-treated rats.

Rat pituitary glands were surgically removed, total RNA was isolated and analysed by quantitative real-time PCR. There was a significant increase of pituitary C5aR expression 24 hours after CLP in animals receiving preimmune IgG, while anti-C5a-treated littermates displayed expression levels comparable with sham animals (Figure 2a). Similar results were found when pituitaries were homogenised and obtained proteins were subjected to Western blot analysis. Protein expression of C5aR in the pituitary was markedly increased in IgG-treated animals 24 hours after CLP, while rats that were administered anti-C5a revealed C5aR protein expression similar to sham controls (Figure 2b). Such patterns of increased C5aR expression in CLP mice have been described in lung, liver, kidney and heart [12].

### C5a-blockade partially reverses cytokine upregulation in the pituitary gland

**Figure 3:**
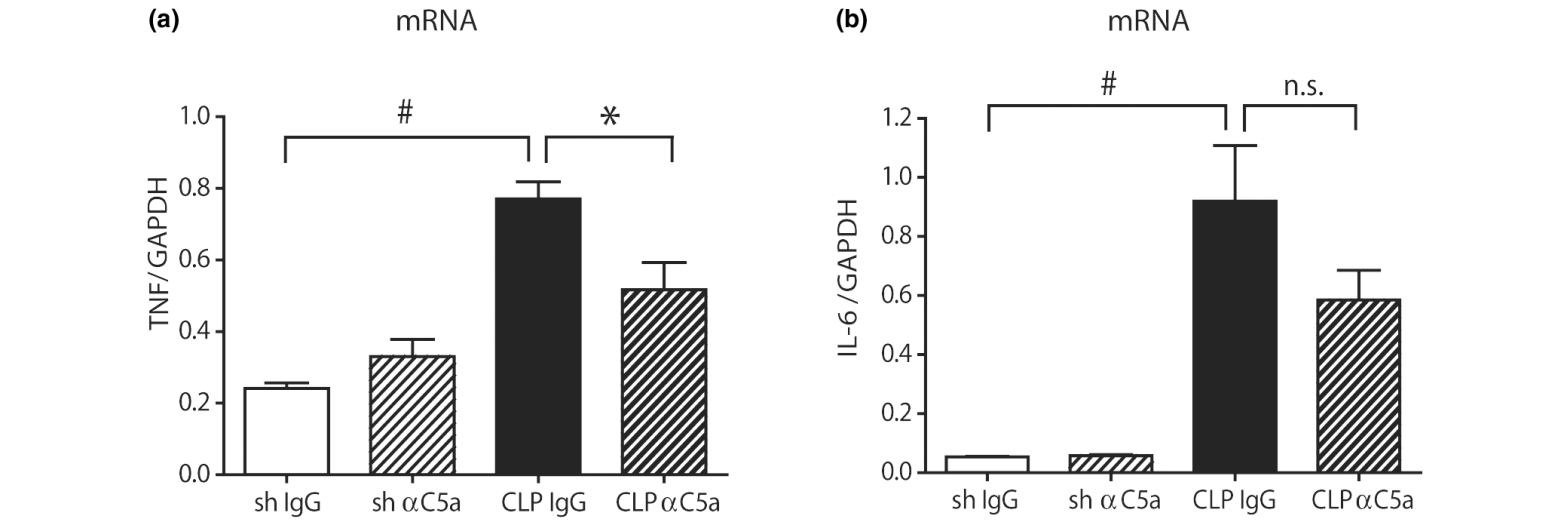
Expression of inflammatory mediators in the pituitary. Pituitary tissue samples were surgically removed, snap-frozen, homogenised and total RNA was extracted. Samples were then analysed by quantitative real-time PCR analysis. Expression of **(a) **TNF and **(b) **IL-6 mRNA in the pituitary 24 hours after sham procedure or caecal ligation and puncture (CLP). Five to seven samples were taken per experimental condition. GAPDH = glyceraldehyde 3-phosphate dehydrogenase. # p < 0.05 between sham and 24 hours CLP animals; * p < 0.05 between IgG-treated and anti-C5a-treated rats.

Following isolation of pituitary total RNA, quantitative real-time PCR was performed for TNF and IL-6. Sham animals treated with either preimmune IgG or anti-C5a displayed similar expression of mRNA for both proinflammatory mediators. However, 24 hours after the CLP procedure, mRNA expression for TNF and IL-6 was substantially increased in IgG-treated rats (Figure 3). Elevated mRNA levels were partially reduced to sham levels when animals were administered anti-C5a immediately after CLP.

### Pituitary dysfunction is reversed by C5a blockade

**Figure 4:**
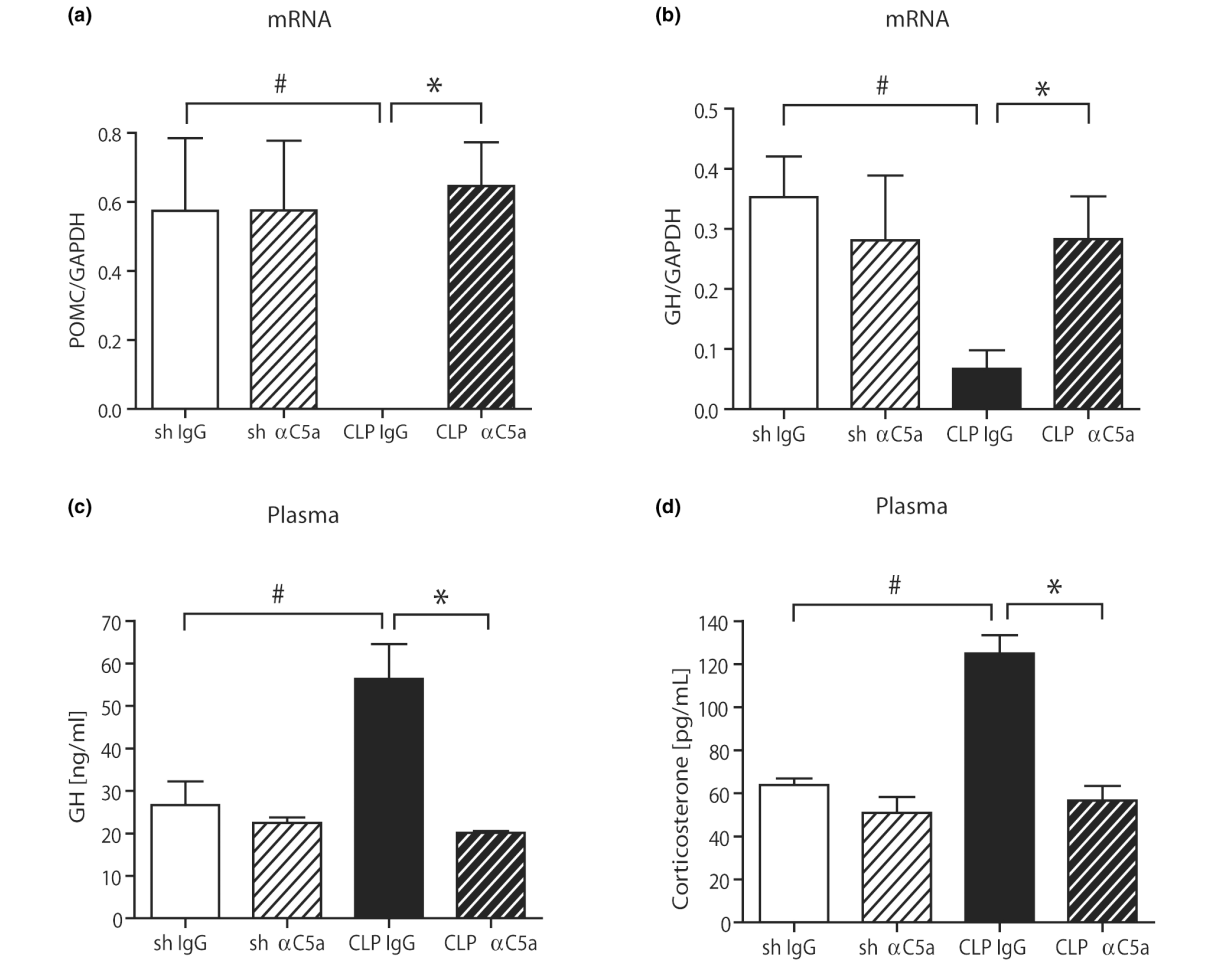
Evaluation of pituitary function after caecal ligation and puncture (CLP). Pituitary tissue samples were removed from four to five mice, snap-frozen, homogenised in Trizol and total RNA was extracted. Assessment of mRNA expression of **(a) **proopiomelanocortin (POMC) and **(b) **growth hormone (GH) 24 hours after CLP or sham operation by real-time PCR. Whole blood samples were drawn at given time-points by puncture of the inferior vena cava, plasma was obtained by centrifugation and subjected to ELISA analysis. Samples were assessed for **(c) **GH or **(d) **corticosterone under identical conditions. For all graphs, there were five to seven samples per experimental condition. GAPDH = glyceraldehyde 3-phosphate dehydrogenase. # p < 0.05 between sham and 24 hours CLP animals; * p < 0.05 between IgG-treated and anti-C5a-treated rats.

Pituitary glands were surgically removed, total RNA was isolated and analysed for POMC and GH by quantitative real-time PCR. mRNA expression for both, POMC and GH was dramatically reduced 24 hours after CLP in IgG-treated rats (Figures 4a,b). Anti-C5a administration completely reversed these changes, resulting in POMC and GH mRNA expression levels equivalent to sham groups. Plasma samples were obtained 24 hours after CLP or sham procedure and subjected to ELISA analysis for GH and corticosterone, the main glucocorticoid of rodents. When compared with sham animals, IgG-treated rats had with significantly increased plasma levels of GH and corticosterone 24 hours after CLP. Again, these changes were reversed by administration of anti-C5a immediately after CLP (Figures 4c,d).

## Discussion

Under physiological conditions, the BBB maintains the cerebral micro-environment by tightly regulating the passage of molecules into and out of the brain to protect the brain from microorganisms and neurotoxic substances. During sepsis, however, blood-borne proinflammatory mediators are released, coincidental with diffuse endothelial activation and subsequent upregulation of vascular adhesion molecule-1 (VCAM-1), intracellular adhesion molecule-1 (ICAM-1), E-selectin on cerebral endothelia [42-45], facilitating adhesion and transmigration of neutrophils and monocytes into the brain tissue. Especially the anaphylatoxin C5a is known to be a strong inducer of ICAM-1, VCAM-1 and various selectins [46-50]. In addition, cerebral endothelia produce IL-1β, TNF and IL-6 [51-53], all of which have been shown to directly induce a disruption of the BBB *in vitro *[54]. Thus, these mediators interact with surrounding brain cells, relaying into the brain inflammatory response and jeopardising the functional integrity of the BBB [55-57]. In the present study, we found that, during experimental sepsis, the antibody-mediated blockade of anaphylatoxin C5a prevented breakdown of the blood-brain barrier, reducing cerebral and pituitary edema formation, as assessed by extravasation of albumin and EB (Figure 1).

Although the CNS itself was traditionally thought to be immunologically privileged, recent studies have demonstrated that the CNS is a rich source of inflammatory mediators, such as cytokines, chemokines and complement components [58-64], and has therefore been termed both 'culprit' and 'victim' during sepsis [57]. Thus, during sepsis, the BBB is exposed to harmful proinflammatory mediators deriving from two different compartments, the brain as well as the systemic circulation. This results in an extrinsic, as well as intrinsic, attack on the BBB, accelerating the deterioration of its barrier function. As described above, we demonstrate significant upregulation of pituitary expression of TNF and IL-6 mRNA following CLP (Figure 3), indicating that the pituitary might be an additional source of cerebral proinflammatory mediators. Blood-derived proinflammatory mediators reach the hypophyseal circulation via the anterior hypophyseal arteries and cytokines can diffuse into the pituitary because these areas are free from CNS BBB [65]. Therefore, we decided that mRNA analysis for TNF and IL-6 might shed a more accurate light on the origin of these proinflammatory mediators.

Resident cells of the brain, such as neurons, astrocytes and microglia, are capable of synthesising essentially all complement proteins, complement regulatory molecules and complement receptors [31,66-70]. It has been reported that the pituitary expresses the complement receptors C3aR, C5aR and C5a-like receptor 2 (C5L2), and that these molecules may contribute to regulation of the immune response [71,72]. We found upregulation of C5aR in the pituitary based on mRNA and protein levels following CLP and reversal of these changes by administration of anti-C5a (Figure 2). Upregulation of C5aR during sepsis has been described in various organs, such as lung, liver, kidney and heart [12]. Such upregulation infers that these tissues may develop highly undesirable outcomes after encounters with C5a.

Sepsis is known to induce an abnormal pituitary response [73]. Hormonal changes in cortisol, mineralocorticoids, thyroid hormones, GH and vasopressin have all been described during sepsis. Although the acute phase of sepsis is characterised by high levels of GH, GH insufficiency is reported in the late stage of sepsis [73,74]. In line with these findings, we have found elevated levels of GH protein 24 hours after CLP, while pituitary mRNA expression is significantly reduced, most likely because of a negative feedback mechanism.

Hypercortisolism during the early stages of sepsis is usually followed by cortisol insufficiency in 60% of septic patients [73]. Significantly increased levels of corticosterone occurred in rats following CLP (Figure 4). In the current study, pituitary POMC mRNA expression was completely abolished during experimental sepsis (Figure 4). POMC is a polypeptide precursor of multiple molecules, including ACTH and melanocyte-stimulating hormones (MSH) α, β and γ [75]. Interestingly, MSH-α has recently emerged as a molecule with potent antiinflammatory effects, which orchestrates descending neurogenic anti-inflammatory pathways and ameliorates the inflammatory response of immune cells [76]. At a molecular level, MSH-α decreases the intracellular production of proinflammatory cytokines and chemokines by inhibiting nuclear factor-κB activation and reduces cellular expression of VCAM-1, ICAM-1 and E-selectin [76]. During human sepsis, plasma concentrations of MSH-α have been found to be significantly decreased during the early course of the disease and gradually recovered as a function of time [77]. More importantly, its concentrations negatively correlated with plasma concentrations of TNF and IL-1β [77]. Thus, it is tempting to speculate, whether the observed pituitary upregulation of TNF and IL-6 mRNA (Figure 3) is related to the complete absence of POMC expression (Figure 4), which will result in a lack of pituitary MSH-α production with uninhibited proinflammatory activation of pituitary cells.

It remains to be determined if our findings can be extrapolated into humans. Several reports have stressed the disconnection between rodent and human sepsis [78-80], making it difficult to draw definitive conclusions from an experimental study for the clinical setting. Moreover, in the current study, the C5a-neutralising antibody was administered immediately after the CLP procedure. Follow-up studies need to address the effects of delayed anti-C5a treatment, because diagnosis of the sepsis syndrome in patients might be delayed due to several co-morbidities. Thus, it is imperative to address if anti-C5a treatment also reverses BBB and pituitary dysfunction after onset of CLP, which would greatly enhance the therapeutic impact of a potential C5a-blockade in humans.

## Conclusions

We describe amelioration of BBB breakdown and partial reversal of pituitary dysfunction by neutralisation of C5a during experimental sepsis. Similarly, blockade of C5aR has recently been described to reduce the LPS-induced activation in the paraventricular nucleus and the central amygdala [81]. Thus, as we are beginning to gain novel insights into the crucial role of C5a in the development of sepsis-induced BBB dysfunction, we might be able to immunomodulate its detrimental effects and improve the outcome of septic encephalopathy.

## Key messages

• Anti-C5a prevents break-down of the BBB during experimental sepsis.

• The C5aR is robustly upregulated in the pituitary gland during CLP-induced sepsis.

• Pituitary mRNA expression of proinflammatory TNF and IL-6 is upregulated during experimental sepsis.

• Experimental sepsis induces pituitary dysfunction which is ameliorated by a neutralising anti-C5a antibody.

## Abbreviations

ACTH: adrenocorticotropic hormone; BBB: blood-brain barrier; C5aR: C5a receptor; C5L2: C5a like receptor 2; CLP: caecal ligation and puncture; CNS: central nervous system; C_T_: cycle threshold; EB: Evans Blue; ELISA: enzyme immunosorbent assay; GAPDH: glyceraldehyde 3-phosphate dehydrogenase; GH: growth hormone; ICAM: intracellular adhesion molecule; ICU: intensive care unit; Ig: immunoglobulin; IL: interleukin; MSH: melanocyte-stimulating hormone; PBS: phosphate buffered saline; PCR: polymerase chain reaction; POMC: proopiomelanocortin; RIPA: radio immunoprecipitation assay; TBST: Tris-buffered saline Tween-20; TNF: tumour necrosis factor; VCAM: vascular adhesion molecule

## Competing interests

The authors declare that they have no competing interests.

## Authors' contributions

MAF, PFS, MHL, PAW and KI designed the study and supervised the experiments. MAF, DR, AND, LMH and BMT performed the experiments. MAF, PFS, SJM, WRS and KI analysed the data and drafted the manuscript. All authors revised the manuscript for important scientific content, read and approved the final manuscript.
